# cGMP-Dependent Protein Kinase Inhibition Extends the Upper Temperature Limit of Stimulus-Evoked Calcium Responses in Motoneuronal Boutons of *Drosophila melanogaster* Larvae

**DOI:** 10.1371/journal.pone.0164114

**Published:** 2016-10-06

**Authors:** Jennifer L. Krill, Ken Dawson-Scully

**Affiliations:** Department of Biological Sciences, Florida Atlantic University, Boca Raton, Florida, United States of America; EPFL, SWITZERLAND

## Abstract

While the mammalian brain functions within a very narrow range of oxygen concentrations and temperatures, the fruit fly, *Drosophila melanogaster*, has employed strategies to deal with a much wider range of acute environmental stressors. The *foraging* (*for*) gene encodes the cGMP-dependent protein kinase (PKG), has been shown to regulate thermotolerance in many stress-adapted species, including *Drosophila*, and could be a potential therapeutic target in the treatment of hyperthermia in mammals. Whereas previous thermotolerance studies have looked at the effects of PKG variation on *Drosophila* behavior or excitatory postsynaptic potentials at the neuromuscular junction (NMJ), little is known about PKG effects on presynaptic mechanisms. In this study, we characterize presynaptic calcium ([Ca^2+^]_i_) dynamics at the *Drosophila* larval NMJ to determine the effects of high temperature stress on synaptic transmission. We investigated the neuroprotective role of PKG modulation both genetically using RNA interference (RNAi), and pharmacologically, to determine if and how PKG affects presynaptic [Ca^2+^]_i_ dynamics during hyperthermia. We found that PKG activity modulates presynaptic neuronal Ca^2+^ responses during acute hyperthermia, where PKG activation makes neurons more sensitive to temperature-induced failure of Ca^2+^ flux and PKG inhibition confers thermotolerance and maintains normal Ca^2+^ dynamics under the same conditions. Targeted motoneuronal knockdown of PKG using RNAi demonstrated that decreased PKG expression was sufficient to confer thermoprotection. These results demonstrate that the PKG pathway regulates presynaptic motoneuronal Ca^2+^ signaling to influence thermotolerance of presynaptic function during acute hyperthermia.

## Introduction

Insects, as poikilotherms, have adapted the ability to function within a wide range of temperatures because their internal temperature assimilates to the ambient temperature of their environment [1–3]. During exposure to extreme environmental stressors, such as an impermissible high temperature, poikilotherms enter into a protective coma which silences neuronal activity [4] and this behavior promotes the recovery of neuronal function once the stressor is relieved [5,6].

Neuronal activity is dependent on the maintenance of ionic concentration gradients along the neuronal membrane and the loss of this ionic homeostasis is the hallmark of neuronal silencing [4,7]. A common consequence of acute stress exposure that includes hyperthermia and anoxia as well as spreading depression events in *Drosophila* [4,7,8], is an increase in extracellular potassium (K^+^) concentration that occurs simultaneously with the loss of neuronal firing [9]. One pathway that targets downstream K^+^ channels is the NO-cGMP-PKG pathway [10,11]. PKG activity alters K^+^ channel conductance which affects extracellular K^+^ levels ([K^+^]_e_) resulting in influences that include: 1) the time course and severity of ionic homeostatic loss [6], 2) the degree of membrane depolarization [7], 3) the time until nerve conduction failure [8], and 4) the functional recovery of locomotion after the stress is alleviated [6,12]. All of these factors ultimately impact an organism’s ability to survive exposure to an acute stress [10].

The regulation of protective coma onset in the fruit fly, *Drosophila melanogaster*, has been linked to the NO-cGMP-PKG pathway [10,13–15] for a variety of stressors including anoxic [10], oxidative stress [11] and heat [13]. Interestingly, PKG enzymatic activity, encoded by the *foraging* gene, influences the time to coma onset in a similar manner across these stressors. For instance, animals with elevated PKG activity behaviorally exhibit motor failure at lower temperatures than animals with lower PKG activity [13,15]. This behavioral failure has been correlated with synaptic failure measured by excitatory postsynaptic potential (EPSP) responses to stimulation of *in vitro* larval preparations [13]. Whether temperature-induced synaptic failure is a result of presynaptic conduction failure or synaptic transmission machinery failure has not been addressed previously and will be the focus of this work.

While the effects that PKG has on [K^+^]_e_ are beginning to be characterized [4,6], the potential effect on the homeostatic loss of intracellular presynaptic Ca^2+^ dynamics is unknown. [Ca^2+^]_i_ is an indicator of synaptic transmission [16]. A change in K^+^ homeostasis that affects neuronal activity should also affect the changes in intracellular Ca^2+^ dynamics that trigger synaptic transmission. This study examines the effect of PKG modulation on presynaptic Ca^2+^ dynamics in motoneuronal boutons at the *Drosophila* larval NMJ by imaging changes in fluorescence of the genetically-encoded Ca^2+^ indicator, GCaMP3 during an increasing temperature ramp. Using pharmacology and tissue-specific *foraging* RNAi (*for* RNAi) to modulate PKG expression and activity, we attempt to understand on a cellular level, during neuronal failure, how PKG modulation affects synaptic transmission during acute hyperthermia. Our results successfully demonstrate that PKG activity has an effect on presynaptic Ca^2+^ dynamics that mediate variations in thermotolerance.

## Materials and Methods

### Fly stocks

Flies were kept in an incubator at 25°C on a standard 12:12-h light-dark cycle and raised on standard Bloomington cornmeal food. The GAL4-UAS system [17] was employed to genetically target expression of the Ca^2+^ indicator GCaMP3 [18] to motoneurons using the enhancer trap line, w1118; P[w+, OK6::Gal4/CyO] [19,20]. Relative Ca^2+^ dynamics were measured during bouts of acute hyperthermia using the line, w*; P{UAS-GCaMP3.T}attP40 [18]. Tissue-specific inhibition of the PKG pathway was targeted to motoneurons using the max w’; ee; UAS-*for* RNAi line acquired from the Sokolowski Lab (University of Toronto) [21]. The balancer line, w; CyO/Sp; TM6B/rf10 was used in the construction of experimental lines with the following genotypes: +/+;UAS-GCaMP3/OK6-GAL4;+/+, +/+;UAS-GCaMP/OK6-GAL4;UAS-*for* RNAi/+ and +/+;UAS-GCaMP/OK6-GAL4;UAS-*for* RNAi/UAS-*for* RNAi.

### Larval NMJ preparation

To examine changes in bouton Ca^2+^ dynamics during acute hyperthermia, Ca^2+^ levels were measured using the genetically-targeted Ca^2+^ indicator, GCaMP3 [18]. The fluorophore is an altered green fluorescent protein (GFP) that emits light in the presence of Ca^2+^. The GAL4-UAS system [17] was used to target the expression of UAS-GCaMP3 to motoneurons using the driver line OK6-GAL4 [19,20].

The *Drosophila* larval NMJ dissection [22] was performed in Schneider’s insect medium (Sigma-Aldrich, St. Louis, MO) on 3^rd^ instar wandering larvae expressing GCaMP3 in Schneider’s insect medium (Sigma-Aldrich, St. Louis, MO). Briefly, animals were pinned dorsal side up on the dissecting dish with standard insect pins placed squarely between the posterior spiracles and the anterior mouth hooks. A horizontal cut was made anterior to the spiracles and then a longitudinal cut was made up the length of the animal. The body wall muscles were pinned down and the internal organs and fat bodies were removed to expose the nervous system. The segmental nerves were cut and the central nervous system was removed to leave a preparation with presynaptic motoneuronal axons, postsynaptic muscles and perisynaptic glia.

Schneider’s medium was replaced with a hemolymph-like saline, HL3, which was made fresh daily in aliquots of 50mL (1mM CaCl_2_, 20mM MgCl_2_, 5mM KCl, 70mM NaCl, 10mM NaHCO_3_, 5mM BES, 115mM sucrose, 5mM trehalose·2H_2_O, 7mM L-glutamic acid) [23,24]. Normally HL3 saline contains 1.5mM Ca^2+^, however, 1mM Ca^2+^ was used in these experiments because increased [Ca^2+^]_e_ can mimic a protective effect on neuronal firing during acute hyperthermia [25]. L-glutamic acid (7mM) was used to prevent muscle contractions to allow imaging of boutons at the NMJ [26]. All experimental solutions were kept on ice to preserve freshness. Small aliquots kept at room temperature were used for dissection to prevent any preconditioning effects from cold shock.

A single segmental nerve toward the center of the animal was drawn into an extracellular glass electrode pulled and forged to ~12–13μm in diameter so that the segmental nerve completely filled the opening. Stimulus-induced neuronal transmission at the larval NMJ was recorded using epifluorescence where increases in intensity indicate an increase in Ca^2+^ inside boutons on muscles 6 and 7. We sampled type 1b boutons only to eliminate confounding Ca^2+^ data as type 1b and type 1s boutons have different Ca^2+^ dynamics [27]. During data analysis, we selected boutons based on their continued ability to respond as well as their maintenance within the focal plane for the duration of the experiment.

### *In vitro* live cell Ca^2+^ imaging

Ca^2+^ imaging methods were based on previously-developed protocols [26,28]. Relative ΔCa^2+^ was imaged with a Nikon Eclipse FN1 upright microscope for physiology using a Nikon B-2E/C filter cube for GFP excitation at 488nm under a Nikon Fluor 60x water-immersion objective. Images were captured with a DS-Qi1MC camera using NIS-Elements AR Software (Nikon Instruments, Inc.) for analysis. Recordings during temperature ramps were taken with an exposure of 600ms every 2s for 25s and a stimulus pulse of 5V, 40Hz for a duration of 5s. Ca^2+^ levels were also recorded for 10s before the stimulus pulse to acquire baseline Ca^2+^ levels. Recording continued for 10s after the stimulus was removed to demonstrate Ca^2+^ levels could return to baseline with changes in temperature for control and treatment groups. All recordings were taken until repeated failures were observed. Increases in temperature influence threshold and hence, affect neuronal ability to elicit an action potential [29]. To ensure the observed failure of Ca^2+^ dynamics within the bouton were not merely due to action potential failure at 5V, 40Hz, the stimulus injection was increased to 11V, 80-100Hz. RNAi experiments were done with the same microscope and objective using the Nikon C1si Fast Spectral Confocal system. Images were acquired using the same settings in EZC1 software (Nikon Instruments, Inc.) with a complete image scan taking 1s/cycle at 512x512 pixels per inch resolution. The images were then analyzed using Nikon NIS Elements similarly to epifluorescence trials. GCaMP3 was chosen for imaging experiments due to its photostability and reduced bleaching compared to other indicators [18]. Limited sampling at discrete temperatures and time points, also limited the effects of bleaching on Ca^2+^ fluorescence.

### Hyperthermia assay

*In vitro* larval preparations were placed on a Peltier plate to control the temperature of the preparation using a laboratory DC power supply (Tektronix, USA). Temperature of the preparation was monitored by placing a K type thermometer (VWR International, Radnor, PA) into the saline adjacent to the larval NMJ preparation with specificity to the tenth of a degree. All baseline recordings were taken at 22.0°C. For HL3 control recordings, the temperature was increased at a rate of 1°C/2min and changes in stimulus-induced Ca^2+^ dynamics were recorded every 2.5°C until failure was observed. Temperature was held constant during recordings. For all drug trials, an initial control was recorded in HL3 only at 22°C prior to the addition of any drug. Non-stimulus-evoked Ca^2+^ dynamics were monitored for a duration of 5min post drug application to detect any basal ΔCa^2+^ due to the addition of drug alone. Initial stimulus-evoked recordings at 22°C for drug application were taken 5min post application and the same temperature ramp and recording protocols were followed until failure was observed.

### Pharmacological manipulation of the PKG pathway

All pharmacological manipulations were applied to *in vitro* 3^rd^ instar larval preparations in HL3 saline. All pharmacological agents were from Sigma-Aldrich, St. Louis, MO. All experimental concentrations were previously verified to confer stress tolerance in in vitro preparations [11,13,30]. A PKG activator, 40μM 8-Bromoguanosine 3′,5′-cyclic monophosphate sodium salt (8-Br-cGMP) [31] or an irreversibly-bound inhibitor, 50μM Rp-8-Bromo-β-phenyl-1,N2-ethenoguanosine 3′,5′-cyclic monophosphorothioate sodium salt (Rp-8-Br-PET-cGMPS) [32,33] were added to the HL3 saline to test the effects of PKG modulation on thermotolerance.

### Image analysis and data processing

Image analysis was performed using previously described methods [26]. The ability to visually discriminate changes in fluorescence intensity of a monochromatic image is limited so a look-up table (LUT) was applied to enhance images through color modifications, non-destructive to raw data, which assign ranges of the visible light spectrum to changes in fluorescence intensity. By applying a LUT to raw image data, the change in [Ca^2+^]_i_ was visually translated across a continuous range of color where the lowest intensity pixels were represented as black and blue and the highest intensity pixels as red. Pixel intensity is correlated to LUT color values in scale bars to the left of representative monochromatic images where LUTs were applied for visualization. Boutons were selected as regions of interest (ROIs) for each experimental trial. A minimum of three ROIs were selected and maintained during imaging of the preparation throughout the temperature ramp. ROIs were selected from type 1 boutons only and were chosen randomly along the NMJ. ROIs were required to maintain their focus within our acquired images for the duration on the experiment to accurately acquire relative Ca^2+^ changes. Stimulus-induced changes in Ca^2+^ were calculated as changes in pixel intensity using the equation ΔF/F = (F_peak_-F_rest_)/F_rest_ where F_peak_ was the maximum ROI pixel intensity and F_rest_ was the pixel intensity of the initial baseline ROI. Individual ROI changes in Ca^2+^ were then averaged for each image. Images were taken every 2s for the duration of a trial so the ROI averages for each image gave a representative change in Ca^2+^ for a given time point before, during or after stimulation at a specific temperature. Intra-trial average Ca^2+^ changes were then averaged across all trials of the same pharmacological treatment to determine the average change in Ca^2+^ due to stimulation as temperature increased. Data was analyzed in NIS Elements Advanced Research software (Nikon Instruments, Inc.).

### Statistical analysis and data presentation

Data was analyzed using Sigma Plot 11 (Chicago, IL). Raw data with values below 0.01 were considered noise and removed as these changes were not visibly discernable from changes in fluorescence due to variations in the focal plane of the preparation, a phenomenon that was observed at higher temperatures in our experimental ramp. Raw failure temperature data was analyzed by a one-way analysis of variance (ANOVA). Changes in presynaptic Ca^2+^ dynamics were analyzed by two-way ANOVA for both temperature and drug treatment. All ANOVA data was then analyzed using the post hoc Holm-Sidak method for determining interactions between individual groups. Bar graphs represent significance alphabetically where “A” represents the highest mean and subsequent letters denote sequentially lower means. Groups of data that were not significantly different from each other were assigned the same letter. Line graphs represent significant groups (p<0.05) using an asterisk (*). Line and bar graphs show mean values ± SEM.

## Results

### Stimulus-induced Ca^2+^ dynamics at the *Drosophila* NMJ

**Fig 1:**
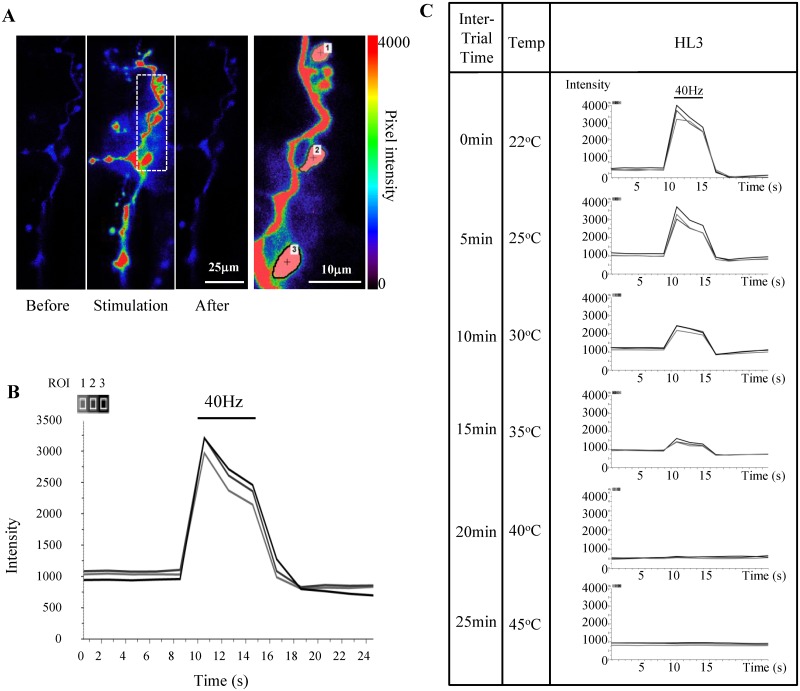
Measuring temperature-dependent changes in stimulus-induced Ca^2+^ dynamics at the *Drosophila* NMJ. (**A**) Ca^2+^ levels at the NMJ before, during and after 5s stimulation. Dotted lines indicate expanded region shown in the inset to the right. Type 1b boutons were randomly selected as regions of interest (ROIs) to measure changes in Ca^2+^ levels during stimulation. (**B**) Changes in Ca^2+^ dynamics measured by changes in pixel intensity for individual ROI over time, before, during and after stimulation. The bar indicates the onset and duration of stimulation. (**C**) Representative traces demonstrate Ca^2+^ response to stimulation decays as temperature increases. Representative ROI traces of a single HL3 preparation show the change in pixel intensity as Ca^2+^ rises and falls in response to 5s stimulation over the time course of the experiment. The bar above the Ca^2+^ curve shows the duration of stimulation. Temperature ramp increases are shown in descending rows and motoneuronal failure can be seen for each treatment as the Ca^2+^ trace flat lines. Data traces were imaged and extracted using NIS Elements (Nikon Instruments, Inc.).

Before addressing the effects that PKG has on presynaptic thermotolerance, stimulus-induced changes in Ca^2+^ dynamics under control conditions were characterized (Fig 1). Bouton Ca^2+^ dynamics at the NMJ show a rise and fall in intensity as observed in the images of the NMJ captured before, during, and after stimulation (Fig 1A). Presynaptic terminal boutons were seen dotted along muscles 6 and 7 of the *Drosophila* 3^rd^ instar larval preparation. A low basal level of Ca^2+^ was observed in the motoneuronal axon as well as the nerve terminals. A subset of boutons was selected as ROIs to assess Ca^2+^ dynamic changes during neuronal firing due to stimulation as temperature increases (Fig 1A, inset). The rise and fall of the intensity of GCaMP3 fluorescence in individual ROIs reflect changes in motoneuronal Ca^2+^ dynamics that coincide with the application and cessation of square pulse stimuli at 40 Hz (indicated by the horizontal bar in Fig 1B). The slight slope seen in the rise and fall of the Ca^2+^ response is artificial and is produced by the 2s increments in time between captured images [34]. The slope of the intensity plateau during stimulation showed a decrease in Ca^2+^ after stimulation onset. This decrease could potentially be caused by a number of factors, including the activation of Ca^2+^ buffering mechanisms within individual boutons after its initial stimulus-induced influx as well as a decrease in the driving force of Ca^2+^ influx over the 5s stimulation duration. Effects of bleaching on Ca^2+^ dynamics were minimal as trace data remained fairly constant in the absence of stimulation (Fig 1C). ROIs within the same trial responded similarly to each other as seen by the close concurrence in the three ROI traces (black, gray and light gray) for a given treatment and temperature (Fig 1C and S1 Fig). The similarity of the ROI Ca^2+^ traces and their reproducibility with additional stimulation provided strong and solid baseline data to assess the effects of temperature on Ca^2+^ dynamics.

### Temperature-dependent effects on Ca^2+^ dynamics

**Fig 2:**
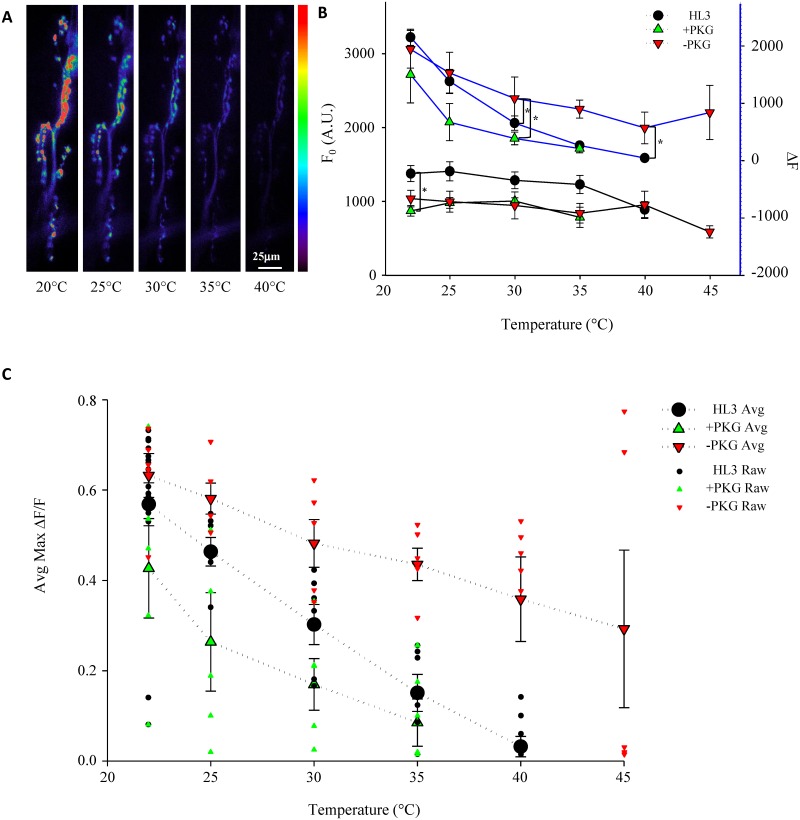
PKG modulation influences temperature-dependent decline in peak Ca^2+^ responses. (**A**) Peak Ca^2+^ fluorescence declined as a result of temperature. Images from a representative HL3 control trial demonstrate the decline in response to stimulation as temperature increased (labeled below image). (**B**) Pharmacological modulation of PKG shows no significant effect on F_0_ or ΔF during hyperthermia (bottom panel and top panel respectively). Average F_0_ Ca^2+^ fluorescence prior to stimulation (bottom panel) is not affected by the addition of PKG activator or inhibitor. For each trial, baseline Ca^2+^ was averaged from ROIs prior to stimulation at each temperature. Baseline Ca^2+^ was then averaged between trials of the same treatment for each temperature in the hyperthermia ramp. A significant difference was seen between +PKG and HL3 at 22°C, however this was the only temperature where a drug treatment had any effect on F_0_ at a given temperature (two-way ANOVA, Holm-Sidak, F_(10,105)_ = 1.770, P = 0.05; * = P<0.05). Average maximum ΔF values were of ROIs showed little difference between treatments at a given temperature (top panel). Average ΔF showed no difference between HL3 and +PKG trials over the entire temperature ramp, however, average ΔF of –PKG was significantly different compared to both HL3 and +PKG ΔF at 30°C and 40°C (two-way ANOVA, Holm-Sidak, F_(10,105)_ = 1.113, P = 0.05; * = P<0.05). (**C**) Temperature-dependent change in average peak Ca^2+^ decline was altered by PKG modulation. Average peak Ca^2+^changes were averaged for ROIs per temperature per trial. Trial averages were then averaged for each condition and temperature to determine the average Ca^2+^ response for drug and control conditions at each temperature. Activation of the PKG pathway with 40μM 8-Br-cGMP (green, n = 5) showed an increased decline in Ca^2+^ dynamics and failed to elicit stimulus-induced Ca^2+^ responses at a much lower temperature than HL3 controls (black, n = 6) or PKG inhibition trials (red, n = 5). Significantly different changes in Ca^2+^ flux between drug treatments for each temperature are shown using brackets with an * (two-way ANOVA, Holm-Sidak, F_(2,105)_ = 36.95, P = 0.05; * = P<0.05).

Changes in stimulus-induced [Ca^2+^]_i_ were measured while increasing temperature. Representative Ca^2+^ responses for an HL3 control preparation during the experimental temperature ramp are shown in Fig 1C. Example traces for preparations with pharmacological agents to either activate (40μM 8-Br-cGMP) or inhibit (50μM Rp-8-Br-PET-cGMPS) the PKG pathway are shown for the same temperature ramp (S1 Fig). Boutons continued to respond to stimulation at higher temperatures similar to room temperature responses (Fig 2C). Although there is a decrease in the peak amplitude of Δ[Ca^2+^]_i_ as temperature rises (Figs 1C and 2A and 2C), this decline is not significant between sequential temperatures within individual treatments (Holm-Sidak, P = >0.050; Fig 2C).

### PKG modulation affects temperature-dependent changes in Ca^2+^ dynamics

To determine the effect PKG has on presynaptic Ca^2+^ dynamics, we next compared HL3 preparations to preparations where either PKG activator or inhibitor were added to the saline. Relative Ca^2+^ dynamics due to stimulation were recorded at room temperature prior to the introduction of heat stress. Although there seems to be a decline in relative Ca^2+^ dynamics when PKG activator was added, there was no significant difference between the stimulus-induced changes in Ca^2+^ at 22°C with PKG activator and those responses in the HL3 control (Holm-Sidak, P = 0.073). Preparations with PKG inhibitor displayed a slight increase in the average peak intensity change, however, this increase was not significantly different from stimulus-induced Ca^2+^responses of HL3 controls at room temperature (Holm-Sidak, P = 0.415). These results demonstrate that PKG modulation does not affect stimulus-induced Ca^2+^ dynamics in the absence of temperature stress (Fig 2C).

As temperature rises, modulation of the PKG pathway influenced stimulus-induced changes of Ca^2+^ in boutons (Fig 2B and 2C). Baseline Ca^2+^ levels (F_0_) are reported along with ΔF values for each treatment as temperature was increased (Fig 2B). Average F_0_ was significantly lower at room temperature for preparations with PKG activation at room temperature compared to HL3 controls (Holm-Sidak, P = 0.003). Ca^2+^ dynamics were recorded at room temperature in HL3 prior to drug application for all drug trials. The average F_0_ in HL3 for PKG activation trials showed lower baseline fluorescence when compared to the average F_0_ at 22°C for HL3 trials (S2A Fig) and the difference in F_0_ in PKG activation trials at room temperature is likely due to the average lower fluorescence of the animals used and not due to the PKG activator itself. There was no change in F_0_ between initial HL3 recordings at 22°C and after PKG activator was applied for 5 minutes (S2B Fig). Whereas PKG activation had a more significant influence on Ca^2+^ dynamics at 25°C compared to HL3 controls (Holm-Sidak, P = 0.007; Fig 2C), inhibition of the PKG pathway influenced Ca^2+^ responses only at higher temperatures. Although PKG activation only demonstrated a significant change in stimulus-induced Ca^2+^ responses at 25°C compared to HL3 controls (Fig 2C and S1 Fig), the decline in Ca^2+^ response with PKG activation is apparent when comparing the ultimate failure temperature of stimulus-induced responses. PKG activation resulted in failure to elicit a response at a lower temperature compared to HL3 controls (Fig 2C).

While PKG activation accelerates the decline of presynaptic Ca^2+^ responses and sensitizes preparations to high-temperature stress, PKG inhibition had the opposite effect and maintained Ca^2+^ responses at much higher temperatures compared to HL3 controls (Fig 2C). Although the effect of PKG inhibition on Ca^2+^ dynamics was not significant at the beginning of the temperature ramp (Holm-Sidak, P = 0.205 at 25°C and P = 0.055 at 30°C), this difference became apparent at higher temperatures and remained significant compared to the control at temperatures equal to and greater than 35°C (Holm-Sidak, P = 0.003 at 35°C, P = <0.001 at 40°C and P = 0.002 at 45°C). The maintenance of stimulus-induced Ca^2+^ dynamics with inhibition of the PKG pathway coincided with the boutons’ ability to continue responding at much higher temperatures compared to the other treatments.

### Pharmacological modulation of PKG influences presynaptic thermotolerance

**Fig 3:**
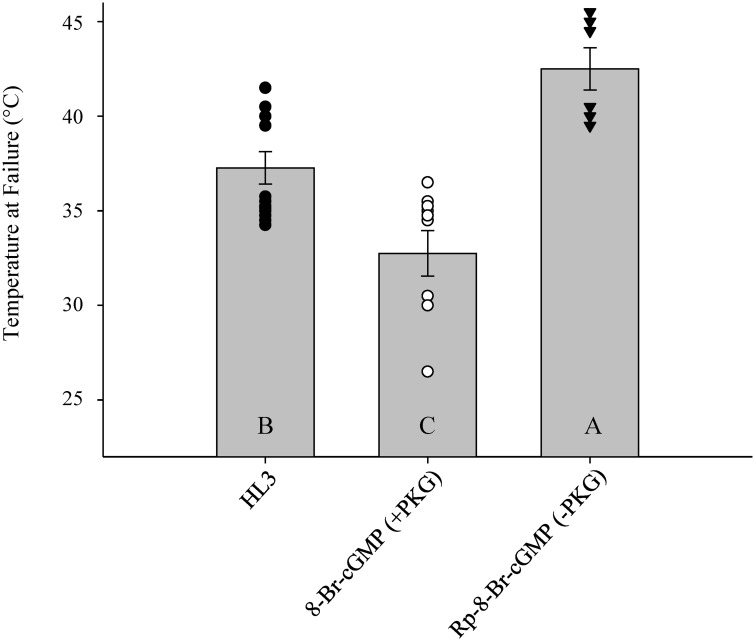
Pharmacological modulation of PKG influences thermotolerance. Average temperature of stimulus-induced Ca^2+^ response failure due to acute hyperthermia was compared for HL3 controls (n = 13), 40μM 8-Br-cGMP PKG activation (n = 10) and 50μM Rp-8-Br-PET-cGMPS PKG inhibition (n = 6). The temperature of Ca^2+^ response failure was significantly different between control HL3, PKG activation and PKG inhibition (Holm-Sidak, df = 2, *P*<0.001). Letters represent significance between groups where A is assigned to the group with the highest mean and bars are represented as mean+/-SEM.

Changes in stimulus-induced Ca^2+^ responses were variable over the increasing temperature ramp across drug treatments and no treatment was consistently significantly different over the entire temperature ramp. To better resolve the effect of PKG modulation on thermotolerance, failure temperature of preparations were compared. Motoneuronal failure was recorded when ROIs no longer exhibited changes in Ca^2+^ as observed with the flat trace at 40°C for HL3, 35°C for PKG activation and 45°C for PKG inhibition in the example preparations (Fig 1C and S1 Fig). All failures were confirmed by increasing stimulus to 11V, 100Hz. The temperature at which stimulus-induced Ca^2+^ dynamics failed for HL3 controls was 37.27±0.86°C (Fig 3). PKG pathway activation reduced the average temperature of motoneuronal failure to 32.75±1.21°C (Fig 3). Attenuation in peak Ca^2+^ amplitude was seen in preparations treated with PKG inhibitor (Fig 2B and 2C and S1 Fig). PKG pathway inhibition allowed the preparation to elicit stimulus-induced Ca^2+^ responses to much higher temperatures of 42.5±1.12°C (one-way ANOVA, F_(2,26)_ = 16.65, P = <0.001; Fig 3). While differences in Ca^2+^ dynamics could not be completely resolved by comparing Ca^2+^ decay, differences in thermotolerance between treatments were apparent when comparing changes to the permissible temperature of motoneuronal function for both PKG activation and inhibition compared to HL3 controls.

### Tissue-specific inhibition of PKG by expressing *foraging* (*for*) RNAi in motoneurons is sufficient for thermotolerance

Pharmacological modulation of PKG demonstrated effects on thermotolerance; however, this drug delivery method could affect other tissues besides motoneurons. Because the *Drosophila* NMJ consists of a presynaptic motoneuron, a postsynaptic muscle and a perisynaptic glial cell, specific modulation of PKG in motoneurons was compared to PKG modulation in other tissues regarding conferring thermotolerance for motoneuronal protection. The GAL4-UAS system was employed to express *for* RNAi to interfere with mRNA translation and prevent PKG expression solely in motoneurons. The OK6 enhancer line was used to drive the expression of UAS-GCaMP3 along with UAS-*for* RNAi. Control and experimental animals were exposed to the same temperature ramp and stimulation parameters as the pharmacological experiments; however, no pharmacological agents were applied to the HL3 saline. Control animals (UAS-GCaMP3/OK6-GAL4; +/+) lacked expression of *for* RNAi whereas experimental groups contained either one (UAS-GCaMP3/OK6-GAL4; UAS-*for* RNAi/+) or two copies of *for* RNAi (UAS-GCaMP3/OK6-GAL4; UAS-*for* RNAi/ UAS-*for* RNAi). The two experimental groups helped to determine the efficacy of the *for* RNAi and OK6 driver lines in knocking down PKG expression. The same protocol of ROI selection and imaging analysis was used as previous pharmacology experiments where ROIs were averaged for each preparation at each temperature and then values from individual preparations were then averaged for each treatment at each temperature.

**Fig 4:**
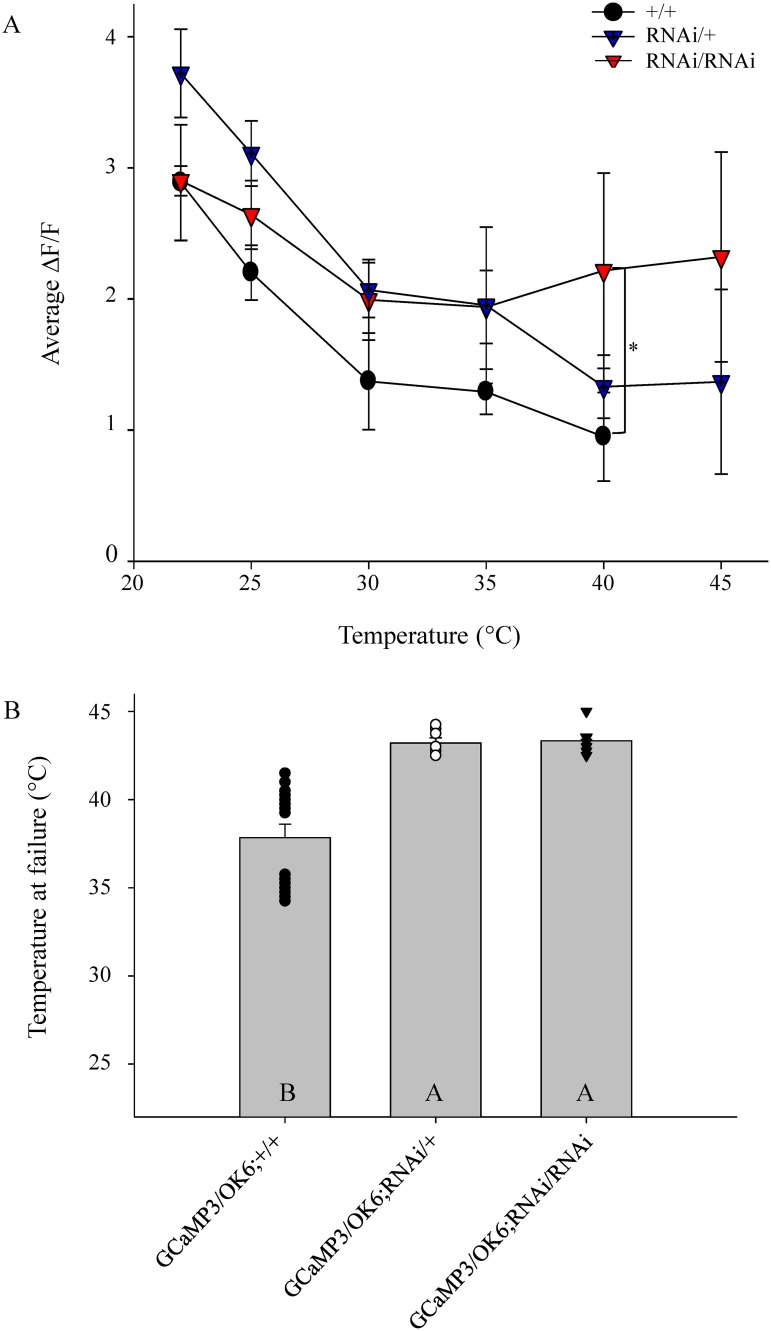
Motoneuronal inhibition of PKG using *for* RNAi is sufficient to confer thermoprotection. (**A**) Temperature-dependent change in average peak Ca^2+^ decay was attenuated by *foraging* knockdown using RNAi. Peak Ca^2+^changes were averaged as outlined in pharmacology experiments. Homozygous expression of for RNAi exhibited significantly different Ca^2+^ dynamics in response to stimulus compared to +/+ controls at 40°C (Holm-Sidak, df = 10, * = P<0.05). (**B**) Expression of *for* RNAi to prevent the expression of PKG solely in motoneurons extended the temperature at which Ca^2+^ dynamics fail. Expression of one copy of *for* RNAi (n = 7) showed no difference in failure temperature from animals with two copies of *for* RNAi (n = 6) (Holm-Sidak, df = 2, *P* = 0.929). Both experimental groups showed an extension in the permissible functional temperature compared to the control group that lacked any copies of *for* RNAi (n = 16) (Holm-Sidak, df = 2, *P*<0.001). Letters represent significance between groups where A is assigned to the highest mean and bars are represented as mean+/-SEM.

No discernable change in Ca^2+^ dynamics was detected at temperatures below 40°C between RNAi expression and controls, however, the temperature-dependent decay in stimulus-induced Ca^2+^ dynamics was attenuated in lines expressing *for* RNAi at temperatures above 40°C. Expression of two copies of *for* RNAi exhibited a significant change in Ca^2+^ dynamics compared to controls at 40°C and this significance was maintained as temperature increased. Heterozygous preparations with only one copy of *for* RNAi demonstrated significantly larger Ca^2+^dynamics compared to controls at 45°C (two-way ANOVA F_(10,186)_ = 0.985, P = <0.05, Fig 4A). The alteration in Ca^2+^ dynamics at high temperatures also influenced the temperature of failure for preparations containing either one or two copies of *for* RNAi. The temperature at motoneuron failure for stimulus-induced Ca^2+^ responses in the control group (GCaMP3/OK6; +/+) was 37.84±0.76°C, much lower than either experimental groups (Fig 4B). Expression of *for* RNAi extended the permissible functional temperature of motoneuronal boutons during acute hyperthermia; motoneurons containing one copy of *for* RNAi failed at 43.21±0.29°C (Fig 4B). An additional copy of *for* RNAi did not extend permissible functional temperatures more than a single copy in animals and temperaturę at failure was 43.33±0.38°C (Fig 4B). Experimental groups were not significantly different from each other, implying that one copy of *for* RNAi was sufficient to knock down PKG expression enough to confer thermoprotection. Both experimental groups expressing *for* RNAi in motoneurons were significantly higher than control groups lacking *for* RNAi expression (one-way ANOVA F_(2,26)_ = 18.85, P = <0.001; Fig 4B).

## Discussion

This investigation demonstrates the ability of PKG to modulate thermotolerance of neuronal function to acute high-temperature exposure. Herein, it was demonstrated that the ability of a motoneuronal bouton to respond to a stimulus decays as temperature increases (Figs 1C and 2C). Inhibition of PKG attenuates the decay of neuronal Ca^2+^ response due to high-temperature stress and neuronal sensitization to higher temperatures can be achieved with PKG activation (Fig 2C). The pharmacological activation of the PKG pathway caused failure of stimulus-induced Ca^2+^ responses to occur at a much lower temperature on average than controls. Conversely, pharmacological inhibition of PKG enabled motoneurons to respond to stimulation at much higher temperatures compared to controls. Finally, knocking down PKG expression specifically in motoneurons using RNAi was sufficient to confer thermotolerance of motoneuronal boutons at high temperatures as witnessed by their continued ability to elicit Ca^2+^ responses (Fig 4). Diminished PKG expression could not be confirmed using western blot because *for* RNAi expression was targeted to motoneurons and the knockdown of PKG specifically in these cells could not be isolated from the expression of PKG in other tissues and a *for*-RNAi line tagged with GFP was not available. Although the *foraging* knockdown of PKG in motoneurons could not be confirmed using western blot nor fluorescence imaging, the RNAi line was confirmed to reduce *foraging* expression using RT-qPCR (unpublished data, Sokolowski Lab). Previously published experiments using the same *for*-RNAi line demonstrate that flies expressing *for*-RNAi phenocopied allelic sitter variants that have reduced PKG expression in short-term memory performance after sleep deprivation stress [21].

In our present study, suppression of motoneuronal PKG expression using *for*-RNAi resulted in raising the permissible temperature of Ca^2+^ responses to stimulation at the NMJ and phenocopied the pharmacological inhibition of PKG with Rp-8-Br-PET-cGMPS. The stimulus-induced increase in [Ca^2+^]_i_ within boutons triggers neurotransmitter release [35,36]. The ability of motoneuronal boutons to continue eliciting Ca^2+^ responses when a stimulus pulse is applied indicates that the neuron is able to generate an action potential and the bouton is still able to signal to the postsynaptic muscle. Because Ca^2+^ is essential to synaptic transmission [35,36], measuring the Δ[Ca^2+^]_i_ of motoneuronal boutons serves as an indicator of the maintenance of neuronal function as well as synaptic transmission. Glutamate was added to the preparation’s saline to desensitize postsynaptic glutamate receptors and prevent muscle contraction so that focused images could be recorded for the entire trial duration [26]. This enabled fluorescent intensity changes due to Ca^2+^ flux to be accurately acquired. Failure of synaptic transmission at the NMJ, measured by excitatory postsynaptic potentials (EPSPs), has been correlated to behavioral failure of larval locomotion during acute hyperthermia exposure [13]. The rate of EPSP decay during hyperthermia is also modulated by PKG and the temperature-induced decay of EPSPs is attenuated when PKG is inhibited [13]. Hyperthermia-induced EPSP decay and ultimate failure is most likely due to a breakdown in presynaptic mechanisms as similar trends are seen with PKG modulation of thermotolerance on motoneuronal terminals (Fig 2C).

The loss of neuronal firing, and hence synaptic transmission, during exposure to acute stress indicates the entrance into a state of stress-induced coma in most poikilotherms, such as insects. The loss of neuronal function during stress-induced coma onset corresponds to a cellular event, the loss of ionic homeostasis [4]. Surges in [K^+^]_e_ occur in insect ganglia across a barrage of environmental stressors and correlate with neuronal silencing [4]. Oxidative stress [11], ATP depletion [12], spreading depression [6], anoxia [10] and hyperthermia [8,9] all exhibit the same [K^+^]_e_ surge upon neuronal failure. In many cases, a gradual rise in [K^+^]_e_ occurs during stress application until the ultimate loss of K^+^ homeostasis [4,7,37,38]. Similarly, there is a gradual rise in [Ca^2+^]_i_ during hyperthermia [39,40], hypoxia [41], and oxidative stress [42]. This same trend of gradually decreasing Ca^2+^ dynamics with increasing temperature was demonstrated in the present study (Fig 2C). The increase in [K^+^]_e_ alters membrane voltage dynamics that could affect voltage-gated Ca^2+^ influx to gradually decrease the peak average Ca^2+^ response of the bouton as temperature rises.

The loss of ionic homeostasis and neuronal failure that result from acute stress exposure has been linked to protein dysfunction [43] as well as depleted mitochondrial ATP production, which in turn compromises ATP powered homeostatic mechanisms [12,40]. The loss of ionic homeostasis with the ultimate surge of K^+^ due to temperature stress exposure would silence the neuron, prevent any additional firing and, hence, any additional Ca^2+^ influx. Given that K^+^ surges coincide with neuronal functional loss [6,8,9,12] and the PKG pathway has been shown to alter K^+^ channel conductance [44] as well as alter stress-induced K^+^ surge onset [6], PKG could be an effective therapeutic target to modulate thermotolerance. Previous studies have been limited to measuring [K^+^]_e_ of insect ganglia, and although [K^+^]_e_ has not been measured in the intracellular space of peripheral synapses, the loss of neuronal function due to the energy-dependent loss of ionic homeostasis could also regulate peripheral neuronal function as well.

While the source of the [K^+^]_e_ surge is still unknown, it could come from the neuron itself or from closely associated glial cells. Whereas PKG has been shown to affect voltage-gated K^+^ conduction [45], a specific K^+^ channel has not been isolated as the main target of PKG modulation and hence the molecular switch that mediates neuroprotection has yet to be found. Cell culture studies have demonstrated that PKG adjusts neuronal excitability through inward rectifying K(ATP) pumps [46] and K^+^ leak channels [47,48]. Brain slice preparations of cholinergic neurons of the basal forebrain of Wistar rats demonstrate that PKG alters the function of K^+^ leak channels [47] and although the changes in K^+^ channel function were explored with increasing pH, the effect of an environmental stressor, such as anoxia or hyperthermia, was never applied. For both the K^+^ leak channels and K^+^ pumps implicated as targets of PKG modulation [49,50], a change in either could alter the membrane potential of the bouton to alter neuronal excitability and firing capabilities. While PKG modulation appears to have a predominant effect on neuronal function during stress as seen with the drastic differences in Ca^2+^ dynamics at higher temperatures, there is still a slight change in Ca^2+^ dynamics at room temperature (Fig 2C). Differences in spontaneous firing have been recorded at the NMJ for allelic variants of PKG; lower PKG activity increases cellular excitability as well as the probability of spontaneous firing in the absence of any stressors. The slight changes in stimulus-induced Ca^2+^ flux at room temperature (Fig 3A) could be due to the changes in neuronal excitability governed by PKG.

The ability of PKG to alter neuronal function during hyperthermia could also include multiple targets. In addition to altering K^+^ channel function along the outer cell membrane[45], PKG also affects Ca^2+^ handling of the endoplasmic reticulum (ER) by modulating ryanodine receptor function and therefore Ca^2+^ release from the ER [51]. PKG modulation of organelle Ca^2+^ handling around the synaptic zone could contribute to the alteration of neuronal function as temperature is gradually increased as well [52,53]. Regardless of the target of intracellular PKG regulation, the genetic expression of *for* RNAi to inhibit PKG expression in motoneurons demonstrates that PKG modulation of motoneuronal function is sufficient to confer functional protection during acute stress. Previous work has demonstrated that PKG activity mediates thermotolerance of synaptic transmission [13] and the present study demonstrates that PKG manifests changes in presynaptic evoked Ca^2+^ dynamics to alter thermotolerance; however, the contribution of perisynaptic glial cells to the modulation of neuronal function at the NMJ has not been assessed. Glial cells have many of the same K^+^ channels as their presynaptic neuronal partners and play a role in regulating extracellular ionic concentrations at the synapse [54]. The hallmark of coma onset is a surge of [K^+^]_e_ [4], and future work could determine whether glia could be an effective target for PKG-mediated regulation of neuronal protection during acute hyperthermia as well as exposure to other stressors.

Protection of locomotory function is thought to be an important factor in the adaptation and survival of insects [55]. Insects that maintain locomotory function in order to move away from a stressful environment are more likely to survive [55]. Studies of adult *Drosophila* locomotory effects of acute hyperthermic stress exposure have also shown differences in allelic variants of PKG that agree with previous larval behavioral findings [15]. On the other hand, activation of the PKG pathway causes sensitization of neural protection to acute stress but it affords the protection of tissue survival [10]. This outcome would be beneficial for environmental insults, disease conditions and trauma that would otherwise result in neuronal damage if function continued during the stress. By entering into a protective, reversible coma, *Drosophila* and other poikilotherms have adapted mechanisms to endure a wide variety of adverse environmental conditions [10,11,15,56]. The PKG pathway appears to be an adaptive switch that could balance function and survival in extreme conditions. While mammals do not have the ability to function in wide ranges of temperature and oxygen levels, the same molecular pathways exist and have the same potential to modulate neuronal protection to hyperthermic stress [56]. Further characterization of the PKG pathway in *Drosophila* and the translation of potential pathway therapeutics to mammals could provide a novel treatment for the prevention of brain damage in humans, who experience trauma in a wide variety of settings, such as stroke, drowning, febrile seizures, and epilepsy, in addition to hyperthermia.

## Supporting Information

S1 Fig Representative traces demonstrate that PKG modulation alters Ca^2+^ response to stimulation as temperature increases. Columns depict representative ROI traces of a single preparation for a given treatment. Traces show the change in pixel intensity as Ca^2+^ rises and falls in response to 5s stimulation of the time course of the experiment. The bar above the Ca^2+^ curve shows the duration of stimulation. Temperature ramp increases are shown in descending rows and motoneuronal failure can be seen for each treatment as the Ca^2+^ traces flat line. PKG activation (Left Panel) sensitizes preparations to acute temperature stress as demonstrated by the flat lines at a lower temperature. On the other hand, PKG inhibition (Right Panel) confers thermotolerance to the preparation at a much higher temperature compared to PKG activator as well as HL3 controls (Fig 1C). Data traces were imaged and extracted using NIS Elements (Nikon Instruments, Inc.).
(TIFF)

S2 Fig Comparison of F_0_ of pharmacology trials. (**A**) Comparison of HL3 **F**_**0**_ in pharmacology trials reveals differences in **F**_**0**_ between HL3 trials and trials using PKG activator or inhibitor. Control responses to Ca^2+^ were taken at room temperature in HL3 prior to drug application. **F**_**0**_ was significantly lower in HL3 responses in drug trials compared to the Ca^2+^ responses at room temperature of trials using only HL3. (one-way ANOVA, F_(2,10)_ = 6.247, Holm-Sidak P = 0.05). (**B**) Difference in F_0_ between HL3 control recording and initial recording with drug (+/-PKG) at room temperature. There is no difference between F_0_ levels when after the drug has been added to the preparation compared to the initial HL3 recordings. Any changes between **F**_**0**_ are likely due to variations in animals and not the application of PKG activator (Students t-test, P = 0.106) or inhibitor (Students t-test, P = 0.125).
(TIFF)

## References

[1] Heinrich B. Thermoregulation in endothermic insects. Science. 1974;185: 747–756. 10.1126/science.185.4153.747 4602075

[2] Armstrong LE, Stoppani J. Central nervous system control of heat acclimation adaptations: an emerging paradigm. Rev Neurosci. 2002;13: 271–85. 10.1515/REVNEURO.2002.13.3.271 12405229

[3] Newman AEM, Xiao C, Robertson RM. Synaptic thermoprotection in a desert-dwelling Drosophila species. J Neurobiol. 2005;64: 170–180. 10.1002/neu.20132 15818554

[4] Rodgers CI, Armstrong GAB, Robertson RM. Coma in response to environmental stress in the locust: a model for cortical spreading depression. J Insect Physiol. Elsevier Ltd; 2010;56: 980–990. 10.1016/j.jinsphys.2010.03.030 20361971

[5] Wu BS, Lee JK, Thompson KM, Walker VK, Moyes CD, Robertson RM. Anoxia induces thermotolerance in the locust flight system. J Exp Biol. 2002;205: 815–827. 11914390 10.1242/jeb.205.6.815

[6] Armstrong GA, Rodgers CI, Money TG, Robertson RM. Suppression of spreading depression-like events in locusts by inhibition of the NO/cGMP/PKG pathway. J Neurosci. 2009;29: 8225–8235. 10.1523/JNEUROSCI.1652-09.2009 19553462 PMC6666049

[7] Armstrong GAB, Xiao C, Krill JL, Seroude L, Dawson-Scully K, Robertson RM. Glial Hsp70 protects K + homeostasis in the Drosophila brain during repetitive anoxic depolarization. PLoS One. 2011;6 10.1371/journal.pone.0028994 22174942 PMC3236231

[8] Money TGA, Rodgers CI, McGregor SMK, Robertson RM. Loss of potassium homeostasis underlies hyperthermic conduction failure in control and preconditioned locusts. J Neurophysiol. 2009;102: 285–293. 10.1152/jn.91174.2008 19386751

[9] Robertson RM. Thermal stress and neural function: Adaptive mechanisms in insect model systems. J Therm Biol. 2004;29: 351–358. 10.1016/j.jtherbio.2004.08.073

[10] Dawson-Scully K, Bukvic D, Chakaborty-Chatterjee M, Ferreira R, Milton SL, Sokolowski MB. Controlling anoxic tolerance in adult Drosophila via the cGMP-PKG pathway. J Exp Biol. 2010;213: 2410–2416. 10.1242/jeb.041319 20581270 PMC2892421

[11] Caplan SL, Milton SL, Dawson-Scully K. A cGMP-dependent protein kinase (PKG) controls synaptic transmission tolerance to acute oxidative stress at the Drosophila larval neuromuscular junction. J Neurophysiol. 2013;109: 649–58. 10.1152/jn.00784.2011 23136350

[12] Rodgers CI, Armstrong GAB, Shoemaker KL, LaBrie JD, Moyes CD, Robertson RM. Stress preconditioning of spreading depression in the locust CNS. PLoS One. 2007;2 10.1371/journal.pone.0001366 18159249 PMC2137934

[13] Dawson-Scully K, Armstrong GAB, Kent C, Robertson RM, Sokolowski MB. Natural variation in the thermotolerance of neural function and behavior due to a cGMP-dependent protein kinase. PLoS One. 2007;2: e773 10.1371/journal.pone.0000773 17712421 PMC1945089

[14] Milton SL, Dawson-scully K. Alleviating brain stress: what alternative animal models have revealed about therapeutic targets for hypoxia and anoxia. Future Neurol. 2013;8: 287–301. 10.2217/fnl.13.12 25264428 PMC4174394

[15] Chen A, Kramer EF, Purpura L, Krill JL, Zars T, Dawson-Scully K. The influence of natural variation at the foraging gene on thermotolerance in adult Drosophila in a narrow temperature range. J Comp Physiol A Neuroethol Sensory, Neural, Behav Physiol. 2011;197: 1113–1118. 10.1007/s00359-011-0672-3 21861180

[16] Dawson-Scully K, Lin Y, Imad M, Zhang J, Marin L, Horne JA, et al Morphological and Functional Effects of Altered Cysteine String Protein at the Drosophila Larval Neuromuscular Junction. Synapse. 2007;61: 790–794. 10.1002/syn.20335 17068777

[17] Brand AH, Perrimon N. Targeted gene expression as a means of altering cell fates and generating dominant phenotypes. Development. 1993;118: 401–415. 8223268 10.1242/dev.118.2.401

[18] Tian L, Hires SA, Mao T, Huber D, Chiappe ME, Chalasani SH, et al Imaging neural activity in worms, flies and mice with improved GCaMP calcium indicators. Nat Methods. 2009;6: 875–881. 10.1038/nmeth.1398 19898485 PMC2858873

[19] Aberle H, Haghighi AP, Fetter RD, McCabe BD, Magalhaes TR, Goodman CS. Wishful thinking encodes a BMP type II receptor that regulates synaptic growth in Drosophila. Neuron. 2002;33: 545–558. 10.1016/S0896-6273(02)00589-5 11856529

[20] Marques G, Bao H, Haerry TE, Shimell MJ, Duchek P, Zhang B, et al The Drosophila BMP type II receptor wishful thinking regulates neuromuscular synapse morphology and function. Neuron. 2002;33: 529–543. 10.1016/S0896-6273(02)00595-0 11856528

[21] Donlea J, Leahy a., Thimgan MS, Suzuki Y, Hughson BN, Sokolowski MB, et al foraging alters resilience/vulnerability to sleep disruption and starvation in Drosophila. Proc Natl Acad Sci. 2012;109: 2613–2618. 10.1073/pnas.1112623109 22308351 PMC3289360

[22] Ramachandran P, Budnik V. Dissection of Drosophila larval body-wall muscles. Cold Spring Harb Protoc. United States; 2010;2010: pdb.prot5469. 10.1101/pdb.prot5469 20679378

[23] Macleod GT, Hegström-Wojtowicz M, Charlton MP, Atwood HL. Fast Calcium Signals in Drosophila Motor Neuron Terminals. J Neurophysiol. 2002;88: 2659–2663. 10.1152/jn.00515.2002 12424301

[24] Stewart BA, Atwood HL, Renger JJ, Wang J, Wu CF. Improved stability of Drosophila larval neuromuscular preparations in haemolymphlike physiological solutions. J Comp Physiol A Neuroethol Sens Neural Behav Physiol. 1994;175: 179–191. 8071894 10.1007/BF00215114

[25] Barclay JW, Robertson RM. Role for calcium in heat shock-mediated synaptic thermoprotection in Drosophila larvae. J Neurobiol. 2003;56: 360–371. 10.1002/neu.10247 12918020

[26] Macleod GT. Imaging and analysis of nonratiometric calcium indicators at the Drosophila larval neuromuscular junction. Cold Spring Harb Protoc. 2012;7: 802–809. 10.1101/pdb.prot070110 22753596

[27] He T, Singh V, Rumpal N, Lnenicka GA. Differences in Ca2+ regulation for high-output Is and low-output Ib motor terminals in Drosophila larvae. Neuroscience. IBRO; 2009;159: 1283–1291. 10.1016/j.neuroscience.2009.01.074 19409207

[28] Dawson-Scully K, Bronk P, Atwood HL, Zinsmaier KE. Cysteine-string protein increases the calcium sensitivity of neurotransmitter exocytosis in Drosophila. J Neurosci. 2000;20: 6039–6047. 20/16/6039 [pii] 10934253 10.1523/JNEUROSCI.20-16-06039.2000 PMC6772598

[29] Dierolf BM, McDonald HS. Effects of temperature acclimation on electrical properties of earthworm giant axons. Z Vgl Physiol. 1969;62: 284–290. 10.1007/BF00395741

[30] Li P, Oparil S, Novak L, Cao X, Shi W, Lucas J, et al ANP signaling inhibits TGF-β-induced Smad2 and Smad3 nuclear translocation and extracellular matrix expression in rat pulmonary arterial smooth muscle cells. J Appl Physiol. 2007;102: 390–398. 10.1152/japplphysiol.00468.2006 17038494

[31] Ruth P, Landgraf W, Keilbach A, May B, Egleme C, Hofmann F. The activation of expressed cGMP-dependent protein kinase isozymes I alpha and I beta is determined by the different amino-termini. Eur J Biochem. 1991;202: 1339–1344. 1662612 10.1111/j.1432-1033.1991.tb16509.x

[32] Petrov AM, Giniatullin AR, Sitdikova GF, Zefirov AL. The role of cGMP-dependent signaling pathway in synaptic vesicle cycle at the frog motor nerve terminals. J Neurosci. 2008;28: 13216–22. 10.1523/JNEUROSCI.2947-08.2008 19052213 PMC6671604

[33] Wei JY, Cohen ED, Yan YY, Genieser HG, Barnstable CJ. Identification of competitive antagonists of the rod photoreceptor cGMP- gated cation channel:??-phenyl-1,N2-etheno-substituted cGMP analogues as probes of the cGMP-binding site. Biochemistry. 1996;35: 16815–16823. 10.1021/bi961763v 8988020

[34] Macleod GT. Synaptic Vesicles: Test for a Role in Presynaptic Calcium Regulation. J Neurosci. 2004;24: 2496–2505. 10.1523/JNEUROSCI.5372-03.2004 15014125 PMC6729477

[35] Llinás R, Steinberg IZ, Walton K. Relationship between presynaptic calcium current and postsynaptic potential in squid giant synapse. Biophys J. 1981;33: 323–51. 10.1016/S0006-3495(81)84899-0 6261850 PMC1327434

[36] Katz B, Miledi R. Further study of the role of calcium in synaptic transmission. JPhysiol(Lond). ENGLAND; 1970;207: 789–801. 10.1113/jphysiol.1970.sp009095 5499746 PMC1348742

[37] Spong KE, Mazzetti TR, Robertson RM. Activity dependence of spreading depression in the locust CNS. J Exp Biol. 2016;219: 626–630. 10.1242/jeb.132456 26747905

[38] Rodgers CI, LaBrie JD, Robertson RM. K+ homeostasis and central pattern generation in the metathoracic ganglion of the locust. J Insect Physiol. 2009;55: 599–607. 10.1016/j.jinsphys.2009.03.004 19482133

[39] Klose MK, Atwood HL, Robertson RM. Hyperthermic preconditioning of presynaptic calcium regulation in Drosophila. J Neurophysiol. 2008;99: 2420–30. 10.1152/jn.01251.2007 18272873

[40] Klose MK, Boulianne GL, Robertson RM, Atwood HL. Role of ATP-dependent calcium regulation in modulation of Drosophila synaptic thermotolerance. J Neurophysiol. 2009;102: 901–13. 10.1152/jn.91209.2008 19474168

[41] Secondo A, Staiano RI, Scorziello A, Sirabella R, Boscia F, Adornetto A, et al BHK cells transfected with NCX3 are more resistant to hypoxia followed by reoxygenation than those transfected with NCX1 and NCX2: Possible relationship with mitochondrial membrane potential. Cell Calcium. Scotland; 2007;42: 521–535. 10.1016/j.ceca.2007.01.006 17343909

[42] Yang K-T, Pan S-F, Chien C-L, Hsu S-M, Tseng Y-Z, Wang S-M, et al Mitochondrial Na+ overload is caused by oxidative stress and leads to activation of the caspase 3- dependent apoptotic machinery. FASEB J Off Publ Fed Am Soc Exp Biol. United States; 2004;18: 1442–1444. 10.1096/fj.03-1038fje 15231730

[43] Zhong N, Beaumont V, Zucker RS. Roles for mitochondrial and reverse mode Na+/Ca2+ exchange and the plasmalemma Ca2+ ATPase in post-tetanic potentiation at crayfish neuromuscular junctions. J Neurosci. United States; 2001;21: 9598–9607. 11739570 10.1523/JNEUROSCI.21-24-09598.2001 PMC6763056

[44] Renger JJ, Yao WD, Sokolowski MB, Wu CF. Neuronal Polymorphism among Natural Alleles of a cGMP-Dependent Kinase Gene, foraging, in Drosophila. J Neurosci. 1999;19: RC28 10493773 10.1523/JNEUROSCI.19-19-j0002.1999 PMC6783051

[45] Renger JJ, Yao WD, Sokolowski MB, Wu CF. Neuronal polymorphism among natural alleles of a cGMP-dependent kinase gene, foraging, in Drosophila. J Neurosci. 1999;19: RC28 10493773 10.1523/JNEUROSCI.19-19-j0002.1999 PMC6783051

[46] Chai Y, Lin Y-F. Dual regulation of the ATP-sensitive potassium channel by activation of cGMP-dependent protein kinase. Pflugers Arch. Germany; 2008;456: 897–915. 10.1007/s00424-008-0447-z 18231807

[47] Toyoda H, Saito M, Okazawa M, Hirao K, Sato H, Abe H, et al Protein kinase G dynamically modulates TASK1-mediated leak K+ currents in cholinergic neurons of the basal forebrain. J Neurosci. 2010;30: 5677–89. 10.1523/JNEUROSCI.5407-09.2010 20410120 PMC6632358

[48] Toyoda H, Saito M, Sato H, Dempo Y, Ohashi A, Hirai T, et al cGMP activates a pH-sensitive leak K+ current in the presumed cholinergic neuron of basal forebrain. J Neurophysiol. 2008;99: 2126–33. 10.1152/jn.01051.2007 18287551

[49] Zilberberg N, Ilan N, Gonzalez-Colaso R, Goldstein SAN. Opening and Closing of KcnkØ Potassium Leak Channels Is Tightly Regulated. The Journal of General Physiology. 2000 pp. 721–734. 11055999 10.1085/jgp.116.5.721 PMC2229483

[50] Yamanaka R, Shindo Y, Hotta K, Suzuki K, Oka K. NO/cGMP/PKG signaling pathway induces magnesium release mediated by mitoKATP channel opening in rat hippocampal neurons. FEBS Lett. Federation of European Biochemical Societies; 2013;587: 2643–2648. 10.1016/j.febslet.2013.06.049 23831575

[51] Suko J, Maurer-Fogy I, Plank B, Bertel O, Wyskovsky W, Hohenegger M, et al Phosphorylation of serine 2843 in ryanodine receptor-calcium release channel of skeletal muscle by cAMP-, cGMP- and CaM-dependent protein kinase. BBA—Mol Cell Res. 1993;1175: 193–206. 10.1016/0167-4889(93)90023-I 8380342

[52] Khatri N, Man H-Y. Synaptic Activity and Bioenergy Homeostasis: Implications in Brain Trauma and Neurodegenerative Diseases. Frontiers in Neurology. 2013 10.3389/fneur.2013.00199 24376435 PMC3858785

[53] Yi M, Weaver D, Hajnoczky G. Control of mitochondrial motility and distribution by the calcium signal: a homeostatic circuit. J Cell Biol. United States; 2004;167: 661–672. 10.1083/jcb.200406038 15545319 PMC2172592

[54] Simard M, Nedergaard M. The neurobiology of glia in the context of water and ion homeostasis. Neuroscience. 2004 pp. 877–896. 10.1016/j.neuroscience.2004.09.053 15561405

[55] Feder ME, Krebs RA. Environmental Stress, Adaptation and Evolution. In: Bijlsma R, Loeschcke V, editors. Basel: Birkh{ä}user Basel; 1997 pp. 155–173.

[56] Armstrong GAB, López-Guerrero JJ, Dawson-Scully K, Peña F, Robertson RM. Inhibition of protein kinase G activity protects neonatal mouse respiratory network from hyperthermic and hypoxic stress. Brain Res. 2010;1311: 64–72. 10.1016/j.brainres.2009.11.038 19945442

