# Supplementary figures and images for: cGMP-Dependent Protein Kinase Inhibition Extends the Upper Temperature Limit of Stimulus-Evoked Calcium Responses in Motoneuronal Boutons of Drosophila melanogaster Larvae
Source: PLoS One. 2016 Oct 6;11(10):e0164114. doi: 10.1371/journal.pone.0164114 (PMC5053426; doi:10.1371/journal.pone.0164114)

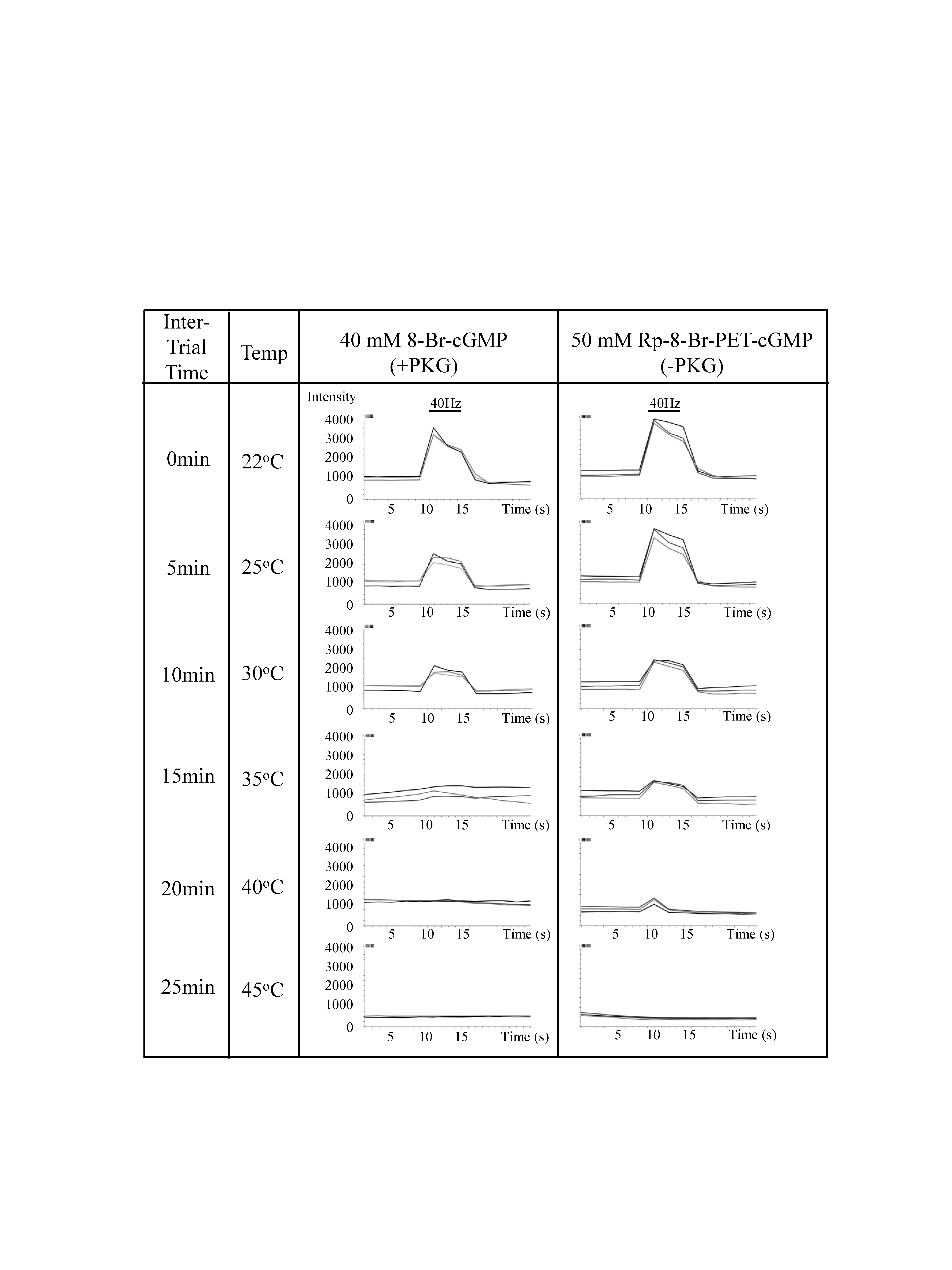

Supplement: S1 Fig — Columns depict representative ROI traces of a single preparation for a given treatment. Traces show the change in pixel intensity as Ca2+ rises and falls in response to 5s stimulation of the time course of the experiment. The bar above the Ca2+ curve shows the duration of stimulation. Temperature ramp increases are shown in descending rows and motoneuronal failure can be seen for each treatment as the Ca2+ traces flat line. PKG activation (Left Panel) sensitizes preparations to acute temperature stress as demonstrated by the flat lines at a lower temperature. On the other hand, PKG inhibition (Right Panel) confers thermotolerance to the preparation at a much higher temperature compared to PKG activator as well as HL3 controls (Fig 1C). Data traces were imaged and extracted using NIS Elements (Nikon Instruments, Inc.). (TIFF) [file pone.0164114.s001.tiff]

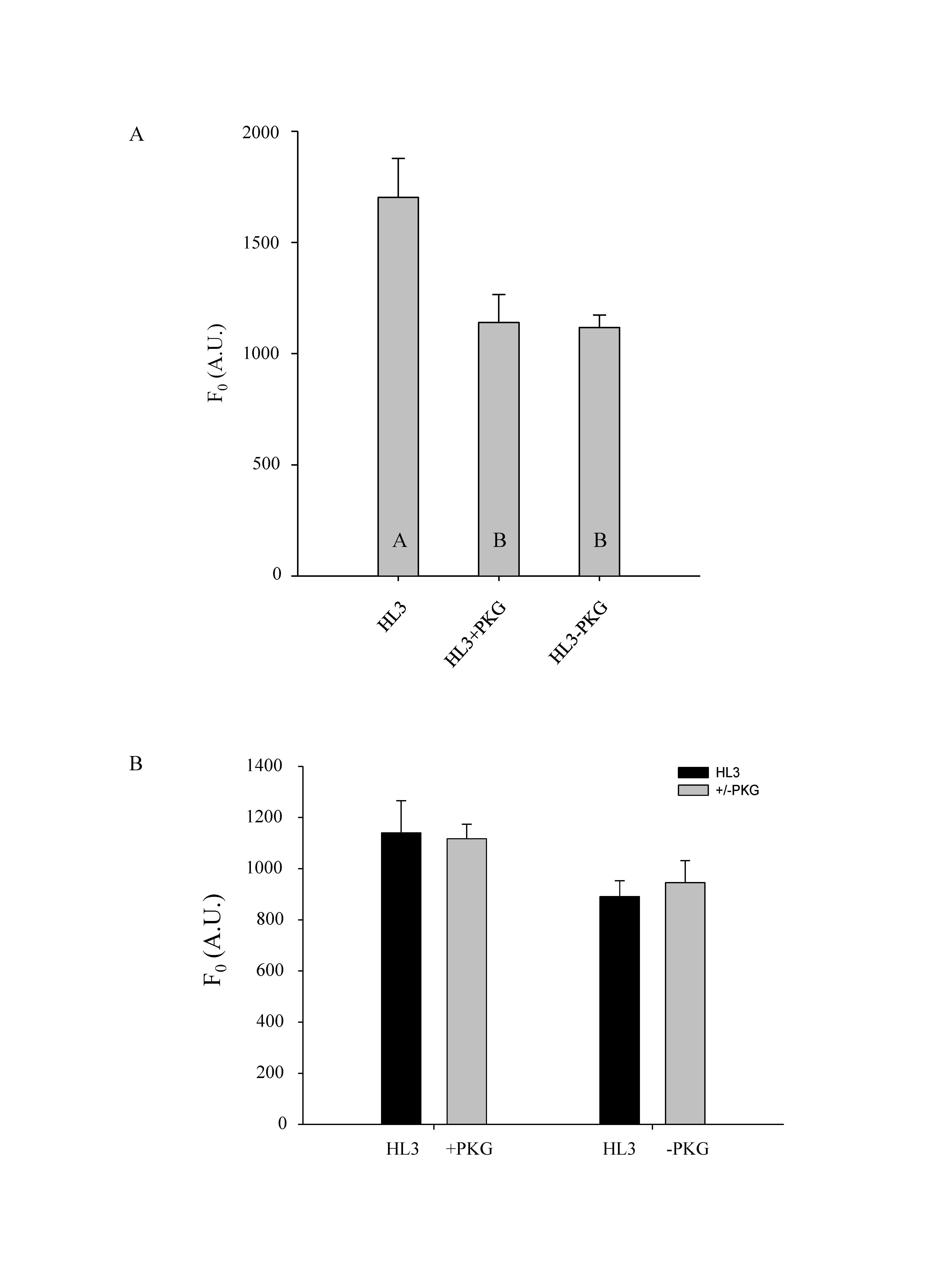

Supplement: S2 Fig — (A) Comparison of HL3 F0 in pharmacology trials reveals differences in F0 between HL3 trials and trials using PKG activator or inhibitor. Control responses to Ca2+ were taken at room temperature in HL3 prior to drug application. F0 was significantly lower in HL3 responses in drug trials compared to the Ca2+ responses at room temperature of trials using only HL3. (one-way ANOVA, F(2,10) = 6.247, Holm-Sidak P = 0.05). (B) Difference in F0 between HL3 control recording and initial recording with drug (+/-PKG) at room temperature. There is no difference between F0 levels when after the drug has been added to the preparation compared to the initial HL3 recordings. Any changes between F0 are likely due to variations in animals and not the application of PKG activator (Students t-test, P = 0.106) or inhibitor (Students t-test, P = 0.125). (TIFF) [file pone.0164114.s002.tiff]
